# Is It Murder?

**Published:** 1872-04

**Authors:** 


### IS IT MURDER?

Some one has sent us a wonderful pamphlet; it is written by a remarkable man—one high in the medical profession, a professor of surgery in Harvard University,—even Prof. H. J. Bigelow.

This pamphlet on "Medical Education in America," is a very queer affair, emanating as it does from one of the great medical teachers of America. It is remarkable for its condemnation of vivisection; asserts that the practice is cruel and useless—that no good has resulted from it, and that it should be frowned down by intelligent medical students. How very strange! We instinctively turn to the title page to learn whether it has been written in this, the nineteenth century, by a distinguished professor of a college that stands at the very head of medical institutions in this country, and find that it is even so. Is it possible that a Harvard professor is not "up" in the modern teachings of nervous physiology, which has revealed to us so many truths, not before observed, and which have been developed entirely through vivisection? Has he read nothing of the action of the various drugs whose powers were only clearly defined through vivisection? Has he seen nothing of the effects that the secretions of the stomach, liver, pancreas, etc., have upon the digestion of food, and which was only learned through vivisection? Has he learned or seen nothing of a thousand facts that have been discovered and only could be learned through vivisection? If it is possible that Prof. Bigelow has seen nor read nothing of this nature, then would there be some excuse for the assertion that no good has ever come of it.

But this is not exactly what we started out to say of Dr. Bigelow's pamphlet. The matter we had in mind was, that portion of it which related to a sort of legal murder, which he earnestly approves, and from his own confession, has extensively practiced.

Although Dr. Bigelow shrinks at the idea of experimenting with the lower orders of animated nature, while under the influence of chloroform, for the purpose of discovering certain physiological facts necessary to be known, for the alleviation of human suffering; yet, he does not hesitate when, in his finite judgment, death is inevitable in a human patient under his care, to deliberately *poison* him, in order to have him "go down to the grave" peacefully, and painlessly! Was ever a proposition more monstrous! Was ever the ennobled calling of the physician more debased!

We do not mean to misrepresent Dr. Bigelow; we have read his pamphlet carefully,—have read it again, and again, trying to convince ourself that he really did not mean what he has written; but if our readers can place a different interpretation upon what here follows, and which we copy literally, as it appears in the pamphlet referred to, we should be glad to know by what manner of logic they arrive at their conclusions. Here is the extract;

"In a surgical practice of twenty-five years, I have never intentionally given a patient, unless by his own choice, any unnarcotized pain, *nor have I allowed a patient to die a death of pain, when opium would lull him into his long sleep.* I share the responsibility of this with the surgeon who walked about the battlefield, distributing morphia to the hopelessly wounded, and with the soldier of Ambrose Parè, *who did more.* It has been my lot to see a friend, at the end of a painful and hopeless malady, to whom, when the hour of death seemed to be near at hand, I had given the morphine largely, twice awaken with a week's new life, due to eighteen or twenty-four hours' deep sleep and continued exemption from pain."

Although the doctor does not distinctly say, yet he leaves us to infer, that while his friend rallied from two doses of morphia, the third one was successful in its aim, and sent him "to his long sleep, exempt from all pain."

While it is entirely proper and humane for an intelligent physician to prescribe morphia to his patient, for the purpose of soothing pain, and to perhaps, give it to the exclusion of curative remedies, when, in his judgment, the case is hopeless; still, no physician has any right whatever to *poison* his patient, and absolutely murder him, in order to end his suffering, no matter how excrutiating the pain, or how hopeless the prospect of recovery. We take it that this position admits of no argument. Now, every physician of any experience, doubtless, has at some time in his life, been called to see a patient, seriously ill or wounded, in whom there was not the slightest prospect for recovery, the physician freely stating his opinion that the patient must die; notwithstanding the unfavorable condition of affairs, he finds at his next visit he has rallied, and at subsequent ones that his symptoms promised final recovery, which was eventually verified. Had the attending medical adviser given morphine, "to lull him into his long sleep," could the act be characterized as murder, or could it not?

Notwithstanding Dr. Bigelow's vast experience as a physician and teacher, he has no right to consider his opinions infallible; greater men than he have made errors of prognosis, and it does not come within his province to usurp the power belonging only to the Almighty.

We hope Dr. Bigelow will reconsider his pamphlet, and make speedy reparation of the great wrong he has done the medical profession in advancing such abominable and wicked doctrines, and write *another* essay, explanatory of the first, in which he may affirm that he really did not mean to be understood as he has so carelessly written.

